# Inflammatory responses in the initiation of lung repair and regeneration: their role in stimulating lung resident stem cells

**DOI:** 10.1186/s41232-016-0020-7

**Published:** 2016-09-12

**Authors:** Mitsuhiro Yamada, Naoya Fujino, Masakazu Ichinose

**Affiliations:** Department of Respiratory Medicine, Graduate School of Medicine, Tohoku University, 1-1 Seiryo-machi, Aoba-ku, Sendai, 980-8574 Japan

**Keywords:** Inflammation, Tissue-resident stem cells, Progenitor cells, M2 macrophage

## Abstract

The lungs are the primary organs for respiration, the process by which carbon dioxide and oxygen are exchanged. The alveolus, which is the site of gas exchange in the lungs, consists of multiple cell types including alveolar epithelial cells, lung capillary endothelial cells and fibroblasts. Because of their complexity, lung parenchymal cells including epithelial lineage have been thought to have a lower rate of cellular turnover in adult lung. However, accumulating observations suggest that the turnover of parenchymal cells in adult lungs is essential for maintaining homeostasis during the steady state as well as for the repair and regeneration after lung injury. After lung injury by harmful pathogens, inflammation occurs to protect the host. Although excessive inflammation damages lung tissue, inflammatory cells are essential for regeneration because they remove harmful pathogens as well as debris derived from apoptotic and necrotic cells. In addition, subsets of inflammatory cells, especially phagocytic monocytes, produce cytokines and growth factors to resolve inflammation and promote tissue regeneration by stimulating tissue-resident stem cells. Recent advances in the biology of lung-resident stem cells, especially those addressing epithelial lineage, have revealed that there are several cellular populations capable of self-renewal that can differentiate into airway and/or alveolar epithelial cells. A part of these populations does not exist in the steady state but emerges after lung injury, suggesting that signals induced by inflammation may play an important role in initiating the proliferation and differentiation of lung stem or progenitor cells. Understanding the interaction between inflammatory responses and tissue-resident stem cells would help elucidate the pathogenesis of inflammatory lung diseases and promote the discovery of new therapeutic targets.

## Background

The lungs are the primary organs for respiration, the process by which carbon dioxide and oxygen are exchanged. The air enters the trachea from the laryngopharynx and continues further into the right and left bronchi. These bronchi split into secondary and tertiary bronchi as the lobes of the lungs. These finally split into smaller bronchioles until they become the respiratory bronchioles. The respiratory bronchioles supply air through alveolar ducts into the alveoli, which are the main place for exchange of the gases. The alveolus is composed of mixed lineage cells including alveolar epithelial cells, lung capillary endothelial cells and fibroblasts. The alveolus is a functional organ unit that provides both efficient gas exchange and a barrier against the external environment. The total surface area of alveoli in human is approximately 60 m^2^. Because of the complexity of the alveolus, lung parenchymal cells including epithelial cells lining the respiratory tract have a lower rate of cellular turnover in adult lung compared to high turnover organs such as the intestine. However, experiments using calorie restriction of adult rodents showed that starvation induced alveolar destruction [1–4] and refeeding induced alveolar regeneration [4, 5]. Starvation in adult humans also leads to alveolar destruction in the lung, suggesting this phenomenon is conserved in humans. These findings imply that the turnover of the parenchymal cells in adult lungs is also essential to maintain homeostasis during the steady state. Moreover, it has been shown that the lung has tissue-resident stem or progenitor cells for regeneration after lung injury. Disruption of the regenerative capacity supported by these resident stem or progenitor cells can cause lung diseases such as emphysema and lung fibrosis [6, 7]. Therefore, to understand the mechanism of regeneration in the lung after lung injury mainly induced by inflammation is likely to be important for understanding the pathogenesis of lung diseases and finding new therapeutic targets. In this review, we focus on the mechanism by which inflammation is resolved and on the initiation of lung tissue regeneration. We then provide an overview of the behaviours of lung stem or progenitor cells, especially focusing on cells of the lung alveolar epithelial lineage, during inflammation after lung injury.

## Resolution of inflammation and initiation of regeneration: roles of inflammatory cells in lung tissue repair

Inflammation is a nonspecific biological response of tissues to harmful stimuli including pathogens. Inflammation promotes protective responses involving the immune system. Although inflammation is a beneficial and indispensable response to protect an individual organism against both external and internal harmful stimuli, inflammation induces significant injury to cells, tissues and organs. Excess or prolonged inflammatory responses cause a wide variety of acute and chronic diseases in various organs including the lungs. Therefore, the resolution of inflammation is important for the repair and regeneration of the lungs after injury.

During acute lung injury induced by harmful stimuli such as pathogenic bacteria, the acute inflammatory response is characterized initially by the generation of mediators (cytokines, chemokines, etc.) that induce the accumulation of neutrophils in alveoli. The emigration of neutrophils from alveolar capillaries to the airspace impairs the alveolar function by damaging alveolar epithelial cells. Therefore, neutrophil accumulation actually worsens the lung injury during the acute phase [8]. However, the neutrophil accumulation also has a role in the repair and regeneration of the lung epithelium. This reparative function of the neutrophil accumulation is partially due to the clearance of cellular debris from the damaged cells in order to create a new matrix sheet for regeneration of the epithelium [9]. It has been also reported that neutrophils directly activate the response for repair of the epithelium. β-catenin signalling is activated in lung epithelial cells during neutrophil transmigration, via the elastase-mediated cleavage of E-cadherin. This activation of β-catenin signalling promotes epithelial repair [10].

During the acute phase of inflammation in the lungs, the alveoli are filled with debris derived from apoptotic or necrotic cells, apoptotic bodies, harmful foreign materials including pathogens, and microvesicles from inflammatory cells and parenchymal cells. These materials must be removed if the alveoli are to be repaired. The cells important for clearing the debris and apoptotic cells are mononuclear-linage phagocytes including alveolar macrophages and monocytes. In alveoli, alveolar macrophages are the tissue-resident phagocytes that defend against harmful exogenous materials including pathogenic microbes. They have various kinds of receptors to sense harmful agents including pathogens [11, 12]. Alveolar macrophages first initiate a proinflammatory reaction to remove harmful agents and propagate the innate immune response. As well as neutrophils, macrophages are also important inflammatory cells that clear pathogens by releasing toxic mediators including reactive oxygen species and phagocytize pathogens or other inflammatory debris. Proinflammatory macrophages (M1 macrophages) can induce tissue damage through the release of toxic species and enzymes including matrix metalloproteinases. M1 macrophages also enhance lung tissue injury through augmenting neutrophil recruitment by releasing chemokines [13, 14].

On the other hand, macrophages have the ability to change their phenotype to anti-inflammatory and tissue-repairing phenotypes [11, 15], and both resident and recruited macrophages play a significant role in repair and regeneration after lung injury. The engulfment of apoptotic cells, called efferocytosis, is one of the important processes by which macrophages change toward M2 macrophages, which are anti-inflammatory macrophages [16]. Efferocytosis induces a decrease in the release of proinflammatory cytokines and chemokines and augments the production of anti-inflammatory cytokines and growth factors that promote the proliferation of lung parenchymal cells (such as TGF-β, IL-10, VEGF and HGF) [17, 18]. Efferocytosis also reduces the release of nitric oxide by inhibiting its synthesis [18]. M2-derived cytokines, including IL-4 and IL-10, increase the expression of mannose receptor, which augments efferocytosis [19].

**Fig. 1:**
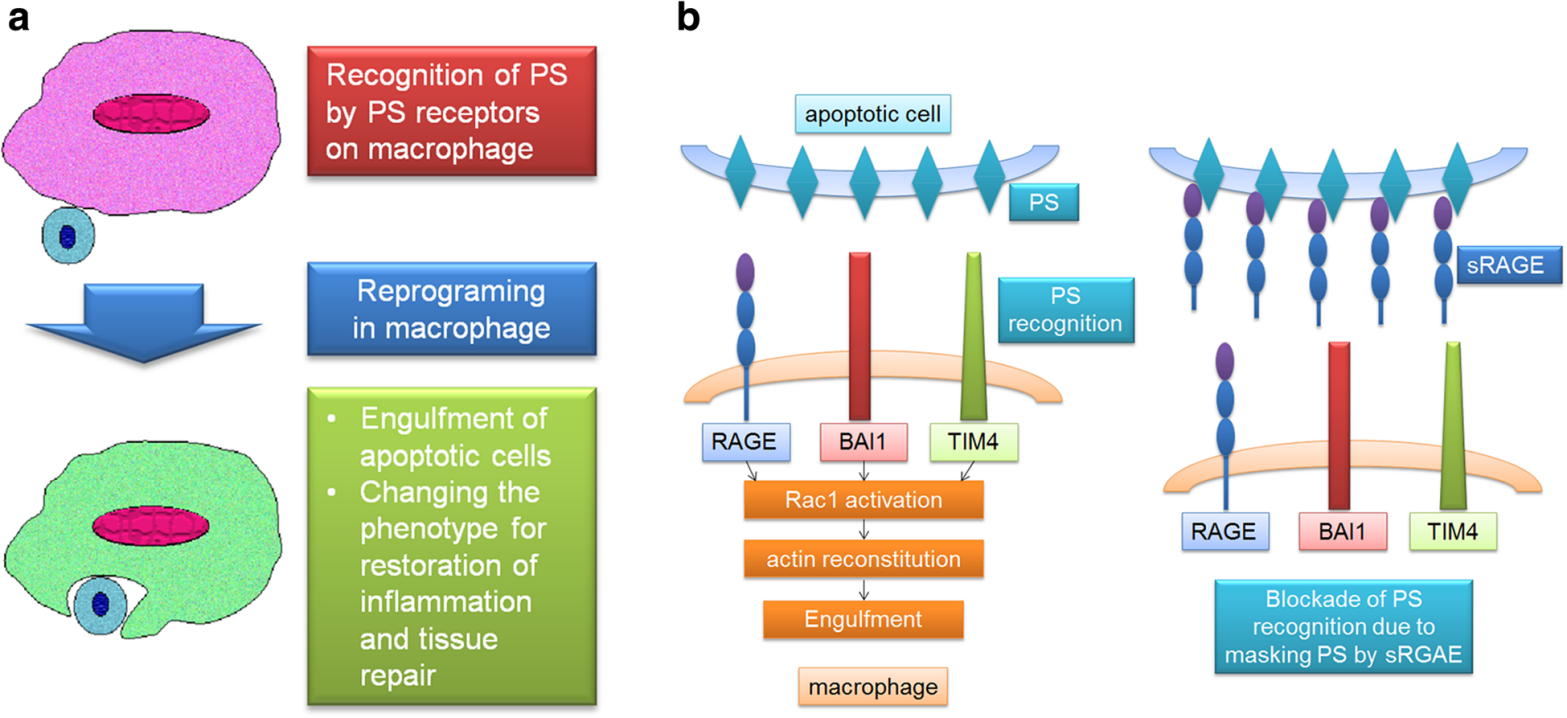
Efferocytosis changes the phenotype of macrophages for tissue repair and regeneration. **a** Recognition of PS induces the engulfment of apoptotic cells as well as the reprograming of macrophages, which results in the conversion of macrophages into these cells for the restoration of inflammation and tissue repair. **b** PS recognition by RAGE. RAGE is a PS receptor, the function of which is similar to that of other PS receptors including BAI1 and Tim-4 (*left*). sRAGE may block PS binding to Ps receptors by masking PS as a decoy receptor

The recognition of phosphatidylserine (PS) structures by mononuclear phagocytes is an important step in the initiation of efferocytosis (Fig. 1a). PS normally exists in the inner cell membrane but rapidly emerges on the cell surface during apoptosis. Several membranous proteins have been identified as receptors that recognize PS. Tim4 is a type I membrane protein that is expressed on phagocytic monocytes including macrophages [20]. It has been shown that Tim4 can bind PS strongly and specifically [20]. A study using Tim4-deficient mice showed that Tim4-deficient macrophages lack the ability to phagocytize apoptotic cells. Other membrane proteins, including CD300 [21–23], phosphatidylserine receptor (PSR) [24], TAM receptor tyrosine kinase Axl [25] and brain-specific angiogenesis inhibitor 1 (BAI1) [26], have been shown to be PS receptors or receptors that promote apoptotic cell engulfment.

We have also identified that the receptor for advanced glycation end products (RAGE) functions as a receptor for PS [27]. RAGE is a membranous protein of the immunoglobulin superfamily [28]. RAGE is expressed on various parenchymal cells, including endothelial cells, and mononuclear phagocytes, including macrophages [29]. It has been reported that RAGE recognizes various ligands including HMGB1, and the binding of RAGE to such ligands is involved in the pathogenesis of various diseases including diabetic vascular disorders, malignancy and inflammation [30]. RAGE is expressed in both a membrane-bound (mRAGE) and soluble form (sRAGE) lacking the transmembrane domain. sRAGE is produced by either the proteolysis of mRAGE or mRNA alternative splicing [31]. sRAGE can work as an inhibitor of RAGE like a decoy receptor for RAGE ligands [32]. We used fluorochrome-labelled sRAGE and found that sRAGE bound to apoptotic thymocytes, indicating that RAGE recognizes and binds to a material expressed on the surface of apoptotic cells. We hypothesized that RAGE could recognize PS and performed a protein–lipid overlay assay, which showed that sRAGE specifically binds to phosphatidylserine. We then performed a surface plasmon resonance analysis to examine the binding affinity of sRAGE to PS and found that the binding response was concentration-dependent with a KD of 0.563 μM. We further performed both FRET analysis and confocal image analysis and found that PS expressed on apoptotic thymocytes binds to full-length mRAGE located in macrophages. Our findings suggest that RAGE can bind specifically to phosphatidylserine.

Using RAGE-deficient mice, we found that alveolar macrophages from RAGE-deficient mice impaired the phagocytic capacity for apoptotic cells, indicating that alveolar macrophages can recognize and phagocytize apoptotic cells through RAGE. We then examined whether sRAGE administration influenced macrophage phagocytosis because sRAGE functions as an endogenous competitive inhibitor of ligand engagement by cell-surface RAGE. sRAGE administration attenuated the phagocytosis of apoptotic cells by wild-type alveolar macrophages, also confirming that RAGE plays a role in the phagocytosis of apoptotic cells. sRAGE also decreased the phagocytic activity of RAGE-deficient alveolar macrophages, possibly suggesting that sRAGE also blocks other types of PS receptor-mediated phagocytosis. We also investigated the intracellular signalling and revealed that Rac1 is activated due to PS recognition through RAGE in alveolar macrophages. These results suggest that RAGE is one of the PS receptors that recognize apoptotic cells. We also examined whether RAGE works for the clearance of apoptotic cells in vivo. We administrated lipopolysaccharide (LPS) into mouse airway, which induced neutrophil migration into the airspace. Once neutrophils migrate, they undergo programmed cell death [33] and are then removed from the airspace through phagocytosis by alveolar macrophages [34]. This resolution process starts in the first few hours after inflammation begins [35]. We observed that more apoptotic neutrophils existed in RAGE-deficient mice during LPS-induced lung injury. RAGE-deficient mice showed a significant increase in the accumulation of inflammatory cells, demonstrating that the deletion of RAGE in macrophages impairs the clearance of apoptotic cells. In summary, the results of our study suggest that RAGE may be one of the PS receptors that recognize apoptotic cells and initiate the clearance of those cells (Fig. 1b). Moreover, sRAGE might inhibit the recognition of PS by cell-surface RAGE and other PS receptors during phagocytosis (Fig. 1b). The balance between sRAGE and mRAGE could modify the phagocytotic activity of macrophages, which might be important in the resolution of inflammation and in tissue regeneration after lung injury. Therefore, RAGE is a PS receptor that may play a role in the resolution of inflammation and in promoting tissue repair and regeneration after lung injury, suggesting that RAGE could be a potential new target for the treatment of human diseases.

## Epithelial cells with the capacities of stem cells and their behaviours in lung injury and inflammation

**Fig. 2:**
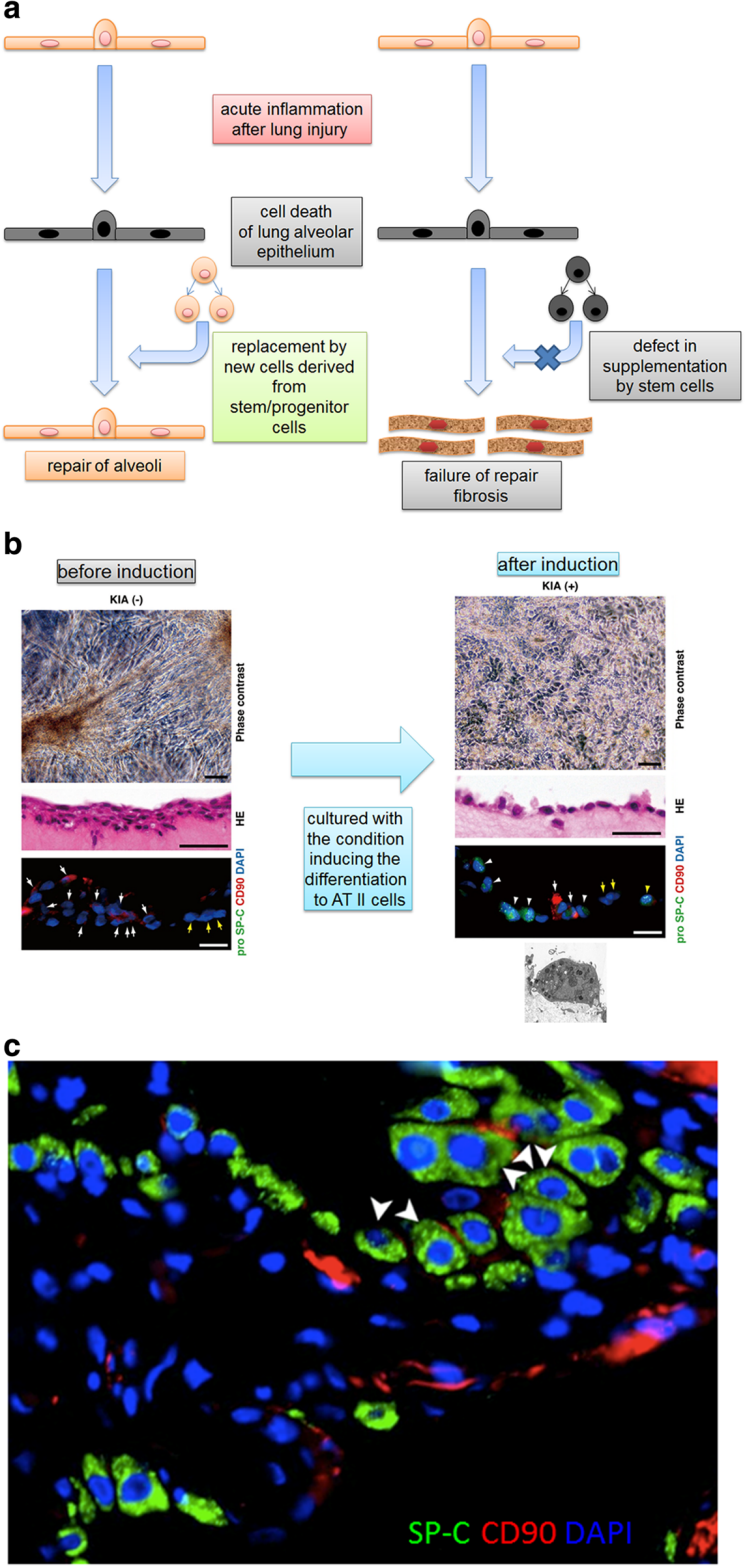
Tissue-resident stem cells for repair and regeneration after lung injury and acute inflammation. **a** Schematic image of the roles of epithelial stem/progenitor cells during lung injury. **b** AEPCs can differentiate to AT II cells in vitro culture. **c** Alveolar epithelial progenitor cells (AEPCs; *arrowheads*) express both CD90 (mesenchymal marker) and pro-SPC (AT II marker) and are localized in the regions of hyperplasia of AT II cells in the lungs of IPF patients

Although inflammatory cells including mononuclear phagocytes contribute to repair and regeneration by clearing debris and producing growth factors, tissue-resident stem cells are critical for tissue repair and regeneration because apoptotic or necrotic parenchymal cells must be replaced by new cells derived from tissue-resident stem cells (Fig. 2a). Recent advances including the analysis of cell fate by in vivo lineage tracing and the identification of new stem cell markers revealed the presence of potential stem cells in the lung, especially in the epithelial cell linage (Table 1).

**Table 1:** Candidate populations as lung epithelial stem cells

Populations	Markers	Cell types into which they can differentiate
Basal stem cells (BSCs)	P63^+^, KRT5^+^, KRT14^+ or −^	BLPCs
Basal luminal precursor cells (BLPCs)	P63+, KRT5^+^, KRT8^+^	ciliated cells, club cells, neuroendocrine cells
Broncho-alveolar stem cells	CC10^+^, pro-SPC^+^	AT II cells, ciliated cells, club cells
ITGα6^+^, ITGβ4^+^ alveolar progenitor cells	CC10^−^, pro-SPC^−^, ITGα6^+^, ITGβ4^+^	AT II cells, club cells
Alveolar type 2 cells (AT II cells)	pro-SPC^+^	AT I cells
Distal alveolar stem cells (DASCs)	P63^+^, KRT5^+^	AT II cells, club cells
Alveolar Epithelial Progenitor cells (AEPCs)	CD90^+^, pro-SPC^+^	AT II cells

The airways of human lungs are covered by a pseudostratified epithelium made of basal cells, secretory cells including CC10^+^ club cells and goblet cells, ciliated cells and neuroendocrine cells. Basal cells are characterized by the expression of P63, nerve growth factor receptor (NGFR) and cytokeratin5 (KRT5) [36, 37]. These cells are capable of self-renewal and can differentiate into ciliated and secretory cells [37, 38]. Some of the basal cells that express KRT14 in the steady state have been shown to be a self-renewing population that maintains the KRT5^+^ basal cell population. A naphthalene-induced injury mouse model showed that this KRT14^+^ KRT5^+^ double-positive cell population is significantly increased [39, 40]. These double-positive cells can directly differentiate into ciliated and secretory cells [41]. Recently, two distinct populations of basal cells were then identified in the adult lung. One is basal stem cells (BSCs) and the other population is referred to as basal luminal precursor cells (BLPCs). Both cell populations express both KRT5 and P63 but do not express KRT14, indicating that KRT14 is not a general marker for identifying stem cell populations [42]. In the steady state, BSCs divide via asymmetric division to produce one new BSC and one BLPC. The BLPCs can further differentiate into neuroendocrine and secretory cells but have a low ability for self-renewal [42]. BLPCs can become distinct from BSCs through KRT8 expression [42]. KRT8 and KRT5 double-positive cells were also identified in mice as progenitor cells during repair and regeneration after injury induced by reactive oxygen species (ROS) and sulphur dioxide (SO_2_) [43]. This SO_2_ injury model showed that P63^+^ basal cell populations divide into other subpopulations prior to the formation of KRT8 and KRT5 double-positive progenitor cells. One of the populations is active Notch2 intracellular domain positive cells, which can differentiate into secretory cells. The other is c-myb positive cells which can differentiate into ciliated cells [44]. This division was not observed in the steady state, suggesting that post-injury mechanisms including inflammatory responses possibly induce different cellular populations of progenitor cells [44]. Tadokoro et al. focused on the potential role of inflammatory cytokine signalling between stem/progenitor cells of a pseudostratified epithelium and their niche [45]. First, they sorted NGFR^+^ basal cells from mouse trachea and performed a clonal 3D organoid assay. They found that IL-6 promoted the differentiation of mouse basal progenitors into ciliated cells, whereas STAT3 inhibitors inhibited the differentiation. The following in vitro experiments also suggested that IL-6/STAT3 signalling promotes the differentiation into ciliated cells by an increase in multicilin and FOXJ1 expression and inhibition of the Notch signalling pathway. To confirm their in vitro findings, they used an SO_2_ injury model and found the activation of STAT3 in basal cells as well as an increase in IL-6 in stromal mesenchymal cells. Using conditional-deficient mice, they found that conditional deletion in basal cells of SOCS3, which is a negative regulator of the STAT3 pathway, resulted in an increase in multiciliated cells after SO_2_ injury, whereas IL-6-deficient mice regenerated fewer ciliated cells after injury. These findings suggest that inflammatory responses possibly function to stimulate stem/progenitor cells for the repair and regeneration of lung tissue.

In the distal lung including the alveolar region, there are candidate stem cells that can differentiate into alveolar epithelial cells. Broncho-alveolar stem cells (BASCs) expressing both CC10 and pro-surfactant protein C (pro-SPC) were identified as cells that can differentiate into both bronchiolar and alveolar epithelial cells in vitro. These cells are located at the broncho-alveolar duct junction (BADJ) [46]. Alveolar type II cells (AT II cells) expressing surfactant protein C were shown to have the ability for self-renewal. Some alveolar type II cells can differentiate into alveolar type I cells (AT I cells) for homeostasis and after injury [47, 48]. In addition to AT II cells, another progenitor subpopulation for alveolar epithelial cells has been identified. These cells are located in both the alveoli and the BADJ and express both α6 and β4 integrin but do not express either CC10 or pro-SPC. During lung injury, these cells proliferate and differentiate into alveolar type II cells and club cells. Distal alveolar stem cells (DASCs) expressing both P63 and Krt5 cells are present in the distal airways during lung injury induced by influenza virus infection. These cells have the ability to differentiate into alveolar epithelial cells [49–51]. KRT5 lineage tracing studies revealed that these cells were not present in the steady state and emerged after injury.

We also isolated colony-forming cells, called alveolar epithelial progenitor cells (AEPCs), which have the ability for self-renewal and the potential to generate alveolar type II cells in vitro (Fig. 2b) [52]. These progenitor cells expressed surface markers of mesenchymal stem cells and surfactant proteins associated with alveolar type II cells, such as CD90 and pro-SPC, respectively (Fig. 2c). Comprehensive expression analyses by microarray revealed that transcripts associated with lung development were enriched in AEPCs compared with bone marrow-mesenchymal stem cells. Histological evaluation indicated that AEPCs were present within alveolar walls in normal lungs. However, these cells significantly increased in the region of AT II cell hyperplasia, suggesting that these cells contribute to alveolar epithelial repair in damaged lungs.

As mentioned above, several cellular populations have been identified as lung epithelial stem/progenitor cells that differentiate into airway and/or alveolar epithelial cells. A part of these populations only emerge during lung injury, suggesting that unidentified inflammatory stimuli induce the emergence or proliferation of lung epithelial stem/progenitor cells. So far, it is still unknown whether these different stem/progenitor cells are really different cells or cells derived from the same origin but phenotypically changed according to circumstances. Further studies including a comprehensive RNA expression assay in a single cell will be necessary to elucidate these issues.

## Conclusions

After lung injury by various harmful stimuli including pathogenic microbes, inflammation occurs for the host defence. Although excessive inflammatory responses are harmful to lung tissue, inflammatory cells are essential for repair and regeneration because these cells are important cleaners that remove harmful pathogens as well as debris derived from apoptotic and necrotic cells. Moreover, inflammatory cells, especially phagocytic monocytes including alveolar macrophages, produce cytokines and growth factors to resolve inflammation and promote tissue repair and regeneration by inducing tissue-resident stem cells. Recent advances in the biology of lung-resident stem cells, especially those of the epithelial lineage, revealed that there are several populations that can self-renew and differentiate into airway and/or alveolar epithelial cells. Interestingly, some of these populations do not exist in the steady state but emerge during inflammation after lung injury, suggesting that signals induced by inflammation may play an important role in initiating the proliferation and differentiation of lung stem/progenitor cells. Further investigation will be needed to understand the interactions between inflammatory responses and tissue-resident stem cells that contribute to lung tissue regeneration in the pathogenesis of inflammatory lung diseases.

## Abbreviations

AEPCs, alveolar epithelial progenitor cells; AT I cells, alveolar type I cells; AT II cells, alveolar type II cells; BADJ, broncho-alveolar duct junction; BASC, broncho-alveolar stem cell; BLPC, basal luminal precursor cells; BSC, basal stem cell; CC10, the club cell 10-kDa protein; FOXJ1, forkhead box protein J1; HGF, hepatocyte growth factor; HMGB1, high-mobility group protein-1; IL-10, interleukin-10; ITG, integrin; KRT14, cytokeratin; KRT5, cytokeratin5; LPS, lipopolysaccharide; mRAGE; membrane-bound receptor for advanced glycation end products; NGFR, nerve growth factor receptor; P63, transformation-related protein 63; Pro-SPC, pro-surfactant protein C; SOCS, suppressor of cytokine signalling; sRAGE, soluble RAGE; STAT, signal transducers and activator of transcription; TGF-β, transforming growth factor-β; Tim4, T cell immunoglobulin and mucin domain-containing molecule 4; VEGF, vascular endothelial growth factor
